# Efficient Dye-Sensitized Solar Cells Based on Nanoflower-like ZnO Photoelectrode

**DOI:** 10.3390/molecules22081284

**Published:** 2017-08-03

**Authors:** Xiaobo Chen, Yu Tang, Weiwei Liu

**Affiliations:** 1 School of New Energy and Electronic Engineering, Yancheng Teachers University, Yancheng 224051, China; wwliu_ciomp@126.com; 2 School of Intelligent Manufacturing, Sichuan University of Arts and Science, Dazhou 635000, China; love-ty11@163.com

**Keywords:** energy storage and conversion, solar energy materials, microstructure

## Abstract

A photoanode material ZnO nanoflower (ZNFs) for efficient dye-sensitized solar cell (DSSC) was prepared. This unique structure can significantly increase the specific surface area and amount of light absorption, leading to a higher short-circuit current density. Furthermore, ZNFs resulted in closer spacing between the nanorods and more direct conduction paths for electrons, leading to higher open-circuit voltage. The overall promising power conversion efficiency of 5.96% was obtained with photoanodes of 8.5 μm thickness. This work shows that ZNFs is an attractive material and has good potential for application in high efficiency ZnO-based DSSCs.

## 1. Introduction

Dye-sensitized solar cells (DSSC) are promising alternatives to conventional solar cells because of their cheap, environmentally friendly, and easy fabrication [1,2]. A porous-structured wide band gap metal oxide film (such as TiO_2_ [3], ZnO [4], and SnO_2_ [5]) as a photoanode is a key component of a typical DSSC, which determines the light-harvesting capability, charge diffusion, and collection efficiency [6]. ZnO has been considered a fascinating alternative photoanode material in DSSCs, since it has a similar electronic band-gap level and higher electron mobility with respect to conventional photoanode material-TiO_2_ [7]. The structure, morphology, crystallinity, and size of ZnO significantly affect the final power conversion efficiency [7,8]. Therefore, various nanostructured ZnO films (such as nanoparticles, nanotubes, nanowires, nanosheets, nanoflakes, hollow spheres, microspheres, and flowers) have been synthesized to enhance PCE in ZnO-based DSCs, which can offer larger surface areas, effective light-scattering centers, or direct electron pathways [7,8,9].

Here, we report on the deposition of ZnO nanoflower (ZNF) photoanodes, which we have developed in an attempt to increase the surface area and ability to reflect and scatter light. An overall power conversion efficiency of 5.96% is obtained by using this highly connected ZNF photoanode with the dye N719, yielding 35.8% enhancement in comparison to DSSCs with ZnO nanoparticle (ZNP) photoanodes.

## 2. Results and Discussion

**Figure 1:**
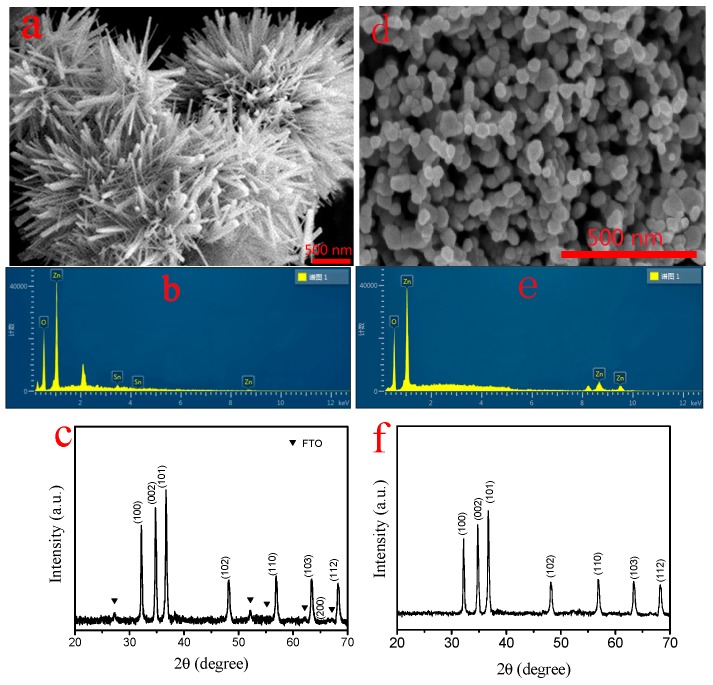
SEM photographs of (**a**) ZNF powder and (**d**) ZNP powder; EDS spectra of (**b**) ZNFs on FTO glass and (**e**) ZNP powder; XRD patterns of (**c**) ZNFs on FTO glass and (**f**) ZNP powder.

Field-emission scanning electron microscopy has been used to investigate the microstructure of the ZnO photoanode, as shown in Figure 1a. The well-defined flower-like morphology could be easily seen. Such a compact flower-like structure of ZnO could benefit the adsorption of dye as well as could provide a direct pathway for the electron transport, which is crucial for light conversion. SEM-coupled energy-dispersive X-ray spectroscopy (EDS) is performed for determining the composition of the grown thin film. Figure 1b shows the EDS of the grown ZNF thin film, in which strong Zn and O peaks with weak Sn peaks are observed. The presence of Zn and O atoms indicates the presence of ZnO, and the Sn peaks originate from the FTO substrate. Figure 1c shows the XRD pattern (XRD, Rigaku) of ZNFs, exhibiting the peaks corresponds to hexagonal wurtzite structure of ZnO (JCPDS 36-1451). Other observed diffraction peaks are associated with FTO substrate. In Figure 1d, it is clear to see that the ZNPs are almost spherical and uniformly distributed with a size around 50 nm. The components and crystallinity of the commercial ZNPs were respectively confirmed by EDS in Figure 1e and XRD pattern in Figure 1e, indicating that the ZNPs were relatively pure hexagonal wurtzite structure ZnO.

**Figure 2:**
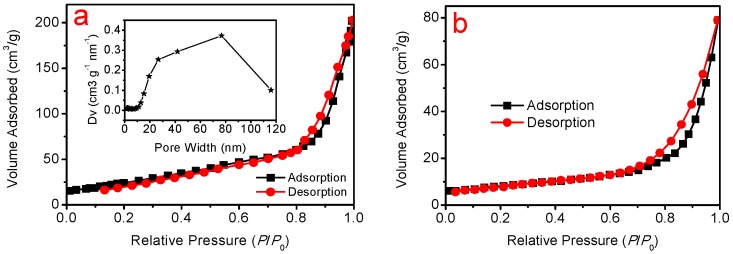
Nitrogen sorption isotherms of the photoanodes. (**a**) The N_2_ adsorption/desorption isotherms and the pore size distribution (inset) of the ZNF photoanode; (**b**) Nitrogen sorption isotherms of the ZNP photoanode.

The porosity of the ZNF is verified by N_2_-sorption isotherm measurement (ASAP 2020, Micromeritics), as shown in Figure 2a. The Brunauer-Emmet-Teller (BET) specific surface area of as-synthesized ZNF photoanode is calculated to be 74 m^2^ g^−1^. The pore size distribution of ZNFs is shown in the inset of Figure 2a. ZNFs exhibit a broad pore size distribution, mostly in the range of 20–100 nm, ensuring that dye could get be absorbed throughout the ZNFs. The BET surface area of a ZNP photoanode is 27 m^2^ g^−1^ (Figure 2b). The thickness of these two photoanode films with similar film thickness was measured to be approximately 8.5 µm, using an optical profilometer (NanoMap-D, AEP Technology) in the optical mode.

**Figure 3:**
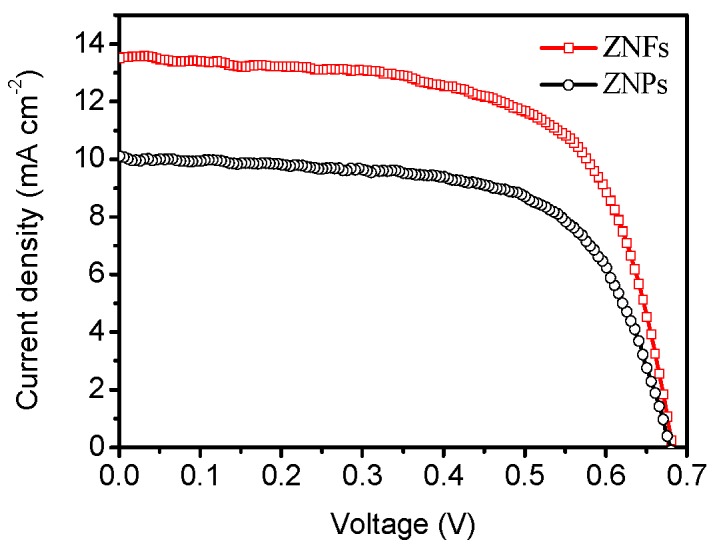
The J-V curves of the DSSCs based on ZNF and ZNP photoelectrodes.

The photovoltaic performance of ZNFs was compared against ZNPs. Figure 3 reports photocurrent density-photovoltage (J-V) measurements under AM 1.5 illumination. The photovoltaic parameters are summarized in Table 1. It can be seen that the two devices have a similar *V*_oc_; because these DSSCs have the same compositions (including same Pt CE and electrolyte), it makes sense that their *V*_oc_ values are close. A comparison of the J-V characteristics of the two photoanode materials indicated that *J*_sc_ was the parameter that contributed to the enhancement of the efficiency for the flower-like ZnO. The *J*_sc_ of ZNF-based-DSSC is about 33.7% larger than the *J*_sc_ of ZNP-based-DSSC. Such a relatively high photocurrent is possibly caused by a higher specific surface area (34 m^2^ g^−1^) and therefore a higher dye loading on the ZNF photoelectrode.

**Figure 4:**
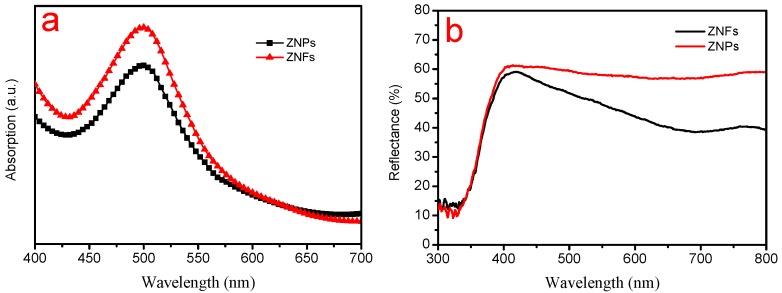
UV/Vis absorption and diffuse reflectance spectra of ZNFs and ZNPs photoelectrodes. (**a**) The UV/Vis absorption spectra of solutions containing dyes detached from the films in NaOH; (**b**) Diffuse reflectance spectra of ZNF and ZNP photoelectrodes before dye adsorption. EIS spectra of the two DSSCs.

The effective dye loading of the photoelectrode was determined from the absorption value for each NaOH-dye solution according to Beer’s law [10], and the UV/Vis absorption spectra are shown in Figure 4a. The dye loading on ZNFs (1.5 × 10^−7^ mol cm^−2^) is higher than ZNPs (1.2 × 10^−7^ mol cm^−2^), which will lead to more photogenerated charge carriers and higher current density for the former. The second factor that contributed to the *J*_sc_ was the enhanced light scattering by the microstructures. A comparison of the diffuse reflectance spectra from ZNF and ZNP photoelectrodes is given in Figure 4b. Compared to the ZNP photoelectrode, the ZNF photoelectrode has higher diffuse reflection capability, thus definitely allowing a higher photocurrent [11].

**Figure 5:**
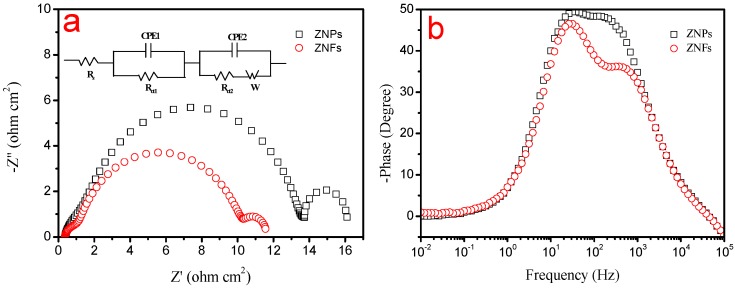
Nyquist plot and Bode phase plot of ZNF and ZNP photoelectrodes. (**a**) Nyquist plot (Inset gives the equivalent circuit used to fit the impedance data) and (**b**) Bode phase plot.

To understand the transport and recombination of photoexcited electrons, electrochemical impedance spectroscopy (EIS) was performed in both the ZNF- and ZNP-based DSSCs by an Electrochemical Workstation. Nyquist plot and Bode phase plot from EIS studies are shown in Figure 5a,b, and the corresponding electrochemical parameters are summarized in Table 2. From the Nyquist EIS plots of DSSCs (Figure 5a), the starting point correlates with the serial resistance (*R*_s_) of the DSSCs. While three semicircles are exhibited in the frequency ranges of 105~103, 103~1, and 1~10^−2^ Hz, corresponding to charger transfer resistance at the counter electrode/electrolyte interface (*R*_ct1_), photoanode/electrolyte interface (*R*_ct2_) [12,13], and Warburg diffusion resistance of I^−^/I_3_^−^ (*W*).The *R*_s_ values have no obvious change, indicating the difference of the photoanode may exert almost no effect on the contact resistance. It is found that there is no obvious deviation for *R*_ct1_, because of the utilization of same Pt CE and I^−^/I_3_^−^ redox electrolyte. According to the method of Adachi et al. [14], the charge-transfer resistance at the ZnO/dye/electrolyte interface (*R*_ct2_) can be obtained from the Nyquist plot. It was found that the *R*_ct2_ for ZNFs was smaller than that for ZNPs, which indicates that electrons are easier to move at the ZNF surface and contribute to the charge transport at the photoanode [15]. The *W* for the ZNFs shows a value lower than that of ZNPs, which reveals that I_3_^−^ can be rapidly reduced to I^−^ to speed up the diffusion of I_3_^−^ [16]. In the Bode phase plot (Figure 5b), the electron lifetime (*τ*) can be estimated using the equation *τ* = 1/(2π*f*_p_), where *f*_p_ is the peak frequency corresponding to the charger transfer process at the photoanode/electrolyte interface. The electrons live longer in the DSSCs based on ZNFs (5.34 ms) compared to that in the DSSCs based on ZNPs (4.84 ms). The enhanced electron lifetime can be attributed to the reduced recombination process, resulting in accelerating electron transfer, increasing electron density, and improving device performance [17]. In short, we can attribute larger surface area, greater ability to reflect and scatter light, and better charge transport properties possessed by ZNFs to their higher conversion efficiency over ZNPs.

## 3. Materials and Methods

ZNFs were synthesized by modifying the procedure as reported by Gupta et al. [18]. Briefly, ZnCl_2_ and NaOH were mixed with a molar ratio of 1:7 and dissolved in pure water. Subsequently, the mixture was placed in a glass reaction vessel and autoclaved by a single mode microwave reactor (2.45 GHz, Discover SP, CEM) at 80 °C for 0.5 h. Finally, the white product was obtained by centrifugation at 7000 rpm for 5 min. The white powder was washed with distilled water and absolute ethanol alternately several times to remove the impurities, and dried in air at 50 °C for 3 h.

Ethyl cellulose, terpineol, and ZNF powder were added into an ethanol solution and stirred to get a paste. Subsequently, the paste was deposited on fluorine-doped tin oxide (FTO) glass substrate by doctor-blade technique. The active area of photoanodes was controlled at 0.16 cm^2^ (0.4 × 0.4 cm). Before dye adsorption, the ZnO films were annealed at 500 °C for 1 h to remove the organic contamination in the paste and improve the interconnecting network. The thermal treatment also increased the crystallinity of the ZnO powder. A conventional ZnO photoanode made from commercial ZNPs (<100 nm in size, Sigma-Aldrich, Shanghai, China) pastes is used as a comparison. The sintered ZnO films were then sensitized by immersing them in a 0.50 mM ethanol solution of N719 dye (purchased from DYESOL LTD, Queanbeyan, Australia) for 24 h at room temperature to obtain dye-sensitized ZnO photoanodes. The commercial Pt CE was used as a counter electrode by maintaining the spacing of approximately 25 μm using a surlyn spacer, which was sandwiched between the electrodes. A drop of electrolyte solution consisting of 10 mM of LiI, 1 mM of I_2_, and 0.1 mM of LiClO_4_ in acetonitrile was injected into the cells. A total of five cells were fabricated using the investigated photoanode to give a representative result.

The morphologies of the surfaces were observed by use of a scanning electron microscopy (SEM, Zeiss Supra 35VP, Berlin, Germany). The crystallographic structure was characterized by X-ray diffraction (XRD) on an X-ray powder diffractometer (X'pert MPD Pro, Philips, Amsterdam, The Netherlands) using Cu Ka radiation (λ = 1.5418 Å). Specific surface areas were measured with a Brunauer–Emmett–Teller (BET) sorptometer (ASAP 2020, Micromeritics, Norcross, GA, USA) using nitrogen adsorption at 77.4 K. The current density-voltage (J-V) curves of the assembled DSSCs were recorded on an Electrochemical Workstation (CHI600E, Shanghai Chenhua Co., Shanghai, China) under irradiation of a simulated solar light (Xe Lamp Oriel Sol3A™ Class AAA Solar Simulators 94023A, Irvine, CA, USA) at a light intensity of 100 mW cm^−2^ (calibrated by a standard Si solar cell). The UV/Vis absorption spectra of solutions containing dyes detached from the films in 3.0 mL H_2_O containing 0.1 M NaOH. The diffuse reflectance spectra of undyed films were investigated by a UV/Vis spectrophotometer (UV-3600, Shimadzu, Tokyo, Japan) equipped with an integrating sphere. The electrochemical impedance spectra (EIS) were recorded by a conventional Electrochemical Workstation (CHI600E, Shanghai Chenhua Co.). EIS scanned from 0.01 Hz to 1 MHz at an ac amplitude of 10 mV was recorded to determine the charge-transfer behaviors, and impedance parameters were extracted by fitting the EIS plots with ZView software according to equivalent circuits derived from a transmission line model.

## 4. Conclusions

In summary, the ZNFs were prepared by hydro-thermal methodascompared withcommercial ZNPs with asize around 50 nm for DSSC application. Consequently, dye loading and light scatting in theZNFs as a photoanodein dye-sensitized solar cells were appreciably enhanced.In addition, ZNFshave a longer electron lifetime and less electron recombinationthan the ZNPs electrode; collectively, theshort-circuit photocurrent density has been greatly increased.The overall conversion efficiency of DSSC with the ZNF photoanode has reached 5.96%, higher than the 4.39% of a ZNP-based-DSSC.

## Figures and Tables

**Table 1:** Summary of photovoltaic and electrochemical properties of the cells.

Photoanode	*V*_oc_ (mV)	*J*_sc_ (mA cm^−2^)	FF (%)	PCE (%)
ZNP	680 ± 1	10.1 ± 0.2	64.0 ± 1	4.39 ± 0.12
ZNF	682 ± 2	13.5 ± 0.1	64.6 ± 1	5.95 ± 0.13

**Table 2:** The electrochemical parameters extracted from the DSSCs with various CEs.

Photoanode	*R*_s_ (Ω cm^2^)	*R*_ct__1_ (Ω cm^2^)	*R*_ct2_ (Ω cm^2^)	W (Ω)	*f*_p_ (ms)	Τ (ms)
ZNP	10.02	3.01	12.75	1.71	32.90	4.84
ZNF	9.89	2.94	9.66	1.35	29.82	5.34
